# Salmonella Infection After Craniotomy

**DOI:** 10.7759/cureus.1566

**Published:** 2017-08-15

**Authors:** Lennox Byer, Caleb Rutledge, Erika Wallender, Joseph A Osorio, Richard Jacobs, Philip V Theodosopoulos

**Affiliations:** 1 School of Medicine, University of California, San Francisco; 2 Department of Neurological Surgery, University of California, San Francisco; 3 Infectious Disease, University of California, San Francisco

**Keywords:** salmonella, meningitis, craniotomy

## Abstract

Salmonella is an uncommon cause of meningitis, especially after neurosurgery. Here, we present a case of Salmonella meningitis after craniotomy, likely due to physical contact with a snake after surgery, with contiguous spread from the patient’s hand to her wound. The purpose of this report is to serve as a reminder that patients undergoing neurosurgery should avoid contact with pets, including snakes and other reptiles, in the postoperative period and practice good hand hygiene.

## Introduction

Patients with Salmonella typically present with symptoms of gastroenteritis, including diarrhea, nausea, vomiting, and abdominal pain [1]. Salmonella meningitis is rare, especially after craniotomy [2]. Here, we present a case of Salmonella meningitis after craniotomy, likely due to physical contact with a snake after surgery, with contiguous spread from the patient’s hand to her wound.

## Case presentation

History

A 24-year-old woman presented to our hospital with a non-enhancing, right frontal lobe mass. She underwent uncomplicated right frontal craniotomy for resection of a World Health Organization (WHO) grade II astrocytoma. Postoperative magnetic resonance imaging (MRI) showed a near-total resection. Her hospital course was uneventful and she was discharged on a three-week dexamethasone taper. However, on postoperative day 13 she developed sudden fever, headache, nausea, and vomiting.

Examination

**Figure 1:**
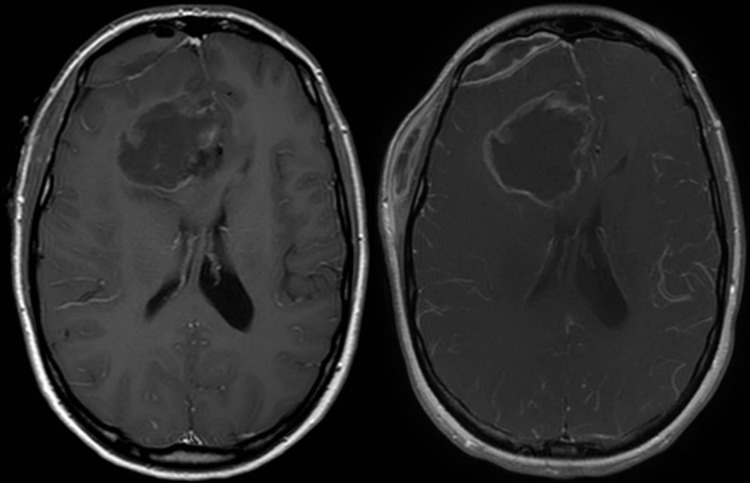
Postoperative T1-weighted MRI with contrast On the left, immediate postoperative axial T1 weighted MRI with contrast. On the right, at presentation axial T1 weighted MRI with contrast showing peripheral enhancement and enlargement of the resection cavity, as well as extra-axial and subgaleal fluid collections with peripheral enhancement. MRI - magnetic resonance imaging.

At an outside emergency room she was febrile with neck stiffness. She was treated empirically with vancomycin and piperacillin and tazobactam before transfer to our hospital. On arrival, an MRI of her brain showed peripheral contrast enhancement and slight enlargement of the resection cavity, an extra-axial fluid collection, and a subgaleal fluid collection with peripheral enhancement (Figure 1). Antibiotics were changed to vancomycin and cefepime. Fluid was aspirated from the subgaleal fluid collection and the gram stain revealed gram-negative rods.

Operation and microbiology findings

The patient was taken to the operating room immediately for washout. Purulent material was noted in the subgaleal and epidural space. The fluid aspirated from the resection cavity appeared to be consistent with cerebrospinal fluid (CSF) with old blood products without any evidence of purulence. Cultures were obtained from the subgaleal and epidural fluid collections as well as from the resection cavity fluid. Cultures from all spaces grew Salmonella group D non typhi. The blood cultures were also positive for Salmonella group D non typhi. 

Postoperative course

Postoperatively, the patient reported that she had handled a pet snake during a family visit five days following her original craniotomy. Upon questioning, she specifically remembered eating fruit without washing her hands following the encounter with the snake. She denied any preceding diarrheal illness, high-risk food intake, travel, or sick contacts. The Infectious Disease service was consulted and she was recommended six weeks of intravenous ceftriaxone followed by oral sulfamethoxazole and trimethoprim.

She was briefly re-hospitalized with fever, rash, and cervical lymphadenopathy after completing intravenous antibiotics and starting sulfamethoxazole and trimethoprim. Her symptoms improved after stopping sulfamethoxazole and trimethoprim, and she was discharged on oral levofloxacin to complete a three-month course of antibiotics. She also required a wound revision for a small dehiscence of her wound after completion of antibiotic therapy and nine months after surgery. The skin was noted to be very thin in the area of the dehiscence without any evidence of erythema. No purulence was noted and intraoperative cultures were negative. Her most recent MRI shows no new enhancement, reduced diffusion, or other evidence of infection.

## Discussion

Salmonella species are flagellated, facultative, anaerobic gram-negative bacilli. Although there are more than 2,000 Salmonella serotypes, clinical laboratories utilize the bacteria’s polysaccharide O-antigen to designate Salmonella into subgroups A through E. Salmonella type D nontyphi includes S. enteriditis, the most commonly identified Salmonella serotype. Foodborne transmission is responsible for the majority of cases, but it may also be transmitted after physical contact with reptiles, including snakes [3].

Salmonella typically usually causes gastrointestinal infections, but it may also cause invasive extraintestinal infections including infections of the central nervous system [4]. The risk factors for invasive, extraintestinal infections include the age and the immune status of the patient. Our patient was immunocompromised by her steroids received postoperatively. In an analysis of 1,316 deaths related to Salmonella infections, most occurred among the immunocompromised and the elderly [5].

In a review of 7,779 cases of Salmonella infection, fewer than 1% had meningitis and most of those infections occurred in infants [1]. Salmonella infection after neurosurgery is even less common. While intracranial Salmonella infections are rare, meningitis, subdural empyema, epidural empyema, and brain abscess have all been reported [4, 6-9].

Reptiles, such as snakes, are commonly colonized with Salmonella both in their digestive tracts and on their skin. Transmission of Salmonella to humans following contact with reptiles is well recognized. Although this was the presumed transmission route in our patient, definitive proof would have required culturing the same serotype of Salmonella from the snake as was found in our patient, something we were not able to obtain.

Traditionally, vancomycin and cephalosporins have been used to treat meningitis empirically after neurosurgery. Cephalosporins, including ceftriaxone and cefepime, cover the majority of Salmonella isolates. Patients treated with third-generation cephalosporins generally have excellent outcomes due to their reliable central nervous system (CNS) penetration.

## Conclusions

Salmonella infection is rare after neurosurgery and few cases have been reported. While most Salmonella infections are foodborne, our patient developed Salmonella meningitis after craniotomy. While bacteremia is associated with invasive Salmonella infections including meningitis, our patient’s exposure to a snake shortly after neurosurgery while being immunocompromised from high-dose steroids is the most likely cause of her infection. Patients undergoing neurosurgery should avoid contact with pets, including snakes and other reptiles, in the postoperative period and practice good hand hygiene.
